# Case of Malformation

**Published:** 1858-05

**Authors:** S. W. Wallace

**Affiliations:** Black Earth, Wis.


### ARTICLE VII.

A gentleman requested me last month to see an infant born the same day, which he said was "busted." I arrived at noon, found the stomach and intestines, except a portion of the rectum external to the abdomen, protruding through an opening about two inches long, by the side of the umbilicus. It died the following morning at ten o'clock. I made a post-mortem examination, found that the opening showed no indications of violence. The œsophagus was attached to the stomach, and seemed to be somewhat stretched. The other parts were *in situ*. S. W. WALLACE, M. D.

*Black Earth, Wis., Dec.* 4, 1858.

